# Sexual experience in premenopausal women with type 1 diabetes: development and validation of a patient-reported outcome measure (PROM)

**DOI:** 10.1016/j.eclinm.2025.103608

**Published:** 2025-10-30

**Authors:** Rahab Hashim, Rita Forde, Judith Parsons, Maddalena De Maria, Davide Ausili, Angus Forbes

**Affiliations:** a Care in Long-Term Conditions, King's College London, London, UK; b University College Cork, Ireland; c Link Campus University, Italy; d University of Milano-Bicocca, Italy

**Keywords:** Type 1 diabetes, Patient-reported outcome measure, Female sexual dysfunction, Sexual experience

## Abstract

**Background:**

Female sexual dysfunction (FSD) is an under-studied problem in diabetes. Current FSD measures do not address diabetes factors (hypoglycaemia, technology and body image) related to women's sexual experiences. This study developed and validated a measure, Female Sexual Experience in Diabetes Type 1 (FSEDiT_1), to assess sexual experience (SE) in premenopausal women with type 1 diabetes (T1D).

**Methods:**

FSEDiT_1 was developed in three phases following the Consensus-based Standards for the selection of health Measurement Instrument (COSMIN) framework. Phase 1. Construct identification - semi-structured interviews with premenopausal women with T1D (n = 18) were conducted between July 2022 and June 2023 to identify constructs associated with SEs, and develop an item bank. Phase 2. Content and structural validity - the items were assessed through focus groups and preliminary testing between August 2023 and May 2024. Phase 3. Psychometric testing (factor analysis, reliability and validity testing) - conducted with participants from a cross-sectional study (n = 429) between June and July 2024. Convergent and discriminant validity were considered using the Diabetes Distress Scale (DDS) and Female Sexual Function Index (FSFI). Test-retest reliability with Intraclass Correlation Coefficients (ICC) was assessed through a sub-sample of participants (n = 44).

**Findings:**

FSEDiT_1 is comprised of five subscales, measuring the impact of diabetes on: initiation of sexual activity (4 items), sexual confidence (6 items), sexual enjoyment (7 items), sexual engagement (6 items) and sexual desire (6 items). Confirmatory factor analysis identified that each of the five subscales had good fit indices (comparative fit index ranged from 0·95 to 0·98 across all the subscales). Test-retest reliability was acceptable with ICC scores of: 0·88 (initiation of sexual activity), 0·87 (sexual confidence), 0·94 (sexual enjoyment), 0·94 (sexual engagement), and 0·93 (sexual desire). FSEDiT_1 demonstrated convergent and discriminant validity.

**Interpretation:**

FSEDiT_1 is a valid and reliable measure of constructs related to SE in premenopausal women with T1D.

**Funding:**

The study is part of a doctoral fellowship funded by the Foundation of European Nurses in Diabetes (FEND). Funding was received from Novo Nordisk Research Foundation to support the transcription of the qualitative interviews in Phase 1.

**Research in Context**

**Evidence before this study**

Prior to conducting this study, we conducted a systematic literature review and meta-analysis to assess the prevalence of sexual dysfunction (SD) in premenopausal women with type 1 diabetes (T1D) and review the tools used to assess female SD (FSD). The review highlighted that the prevalence of SD was threefold higher in premenopausal women with T1D compared to women without diabetes. We identified that all the FSD tools used in the reviewed studies used generic measures for FSD. To our knowledge, there is no FSD tool that addresses the impact of T1D on women's sexual function. To address this knowledge deficit, we decided to develop a diabetes specific measure.

**Added value of this study**

In this study, we present a psychometric evaluation of a novel measure for sexual experience (SE) in women with T1D. It could be used to support observational studies and as an outcome measure for intervention studies. The measure is rooted in the lived experiences of women with T1D, as they identified how diabetes physically, psychologically and socially impacts their sexual activities, engagement and enjoyment.

**Implications of all the available evidence**

Our study has produced the first measure of SE that is specific to premenopausal women with T1D. With this measure, we will be able to provide much more information on the extent of this problem in the diabetes population.

## Introduction

Sexual function is a complex area of human behaviour and is important for mental and physical well-being. Impairments in sexual function for both males and females can cause emotional and relational problems, reducing quality of life.^1^ Clinically, female sexual dysfunction (FSD) receives far less attention compared to male sexual dysfunction (SD) in diabetes.^1^ The prevalence of SD in women with diabetes is much higher compared to those without diabetes due to the physical, psychological, and social impacts of diabetes.^2^^,^^3^ One factor contributing to the under recognition of FSD in this group is the absence of a validated scale specifically to measure SD in the context of diabetes.^1^ A recent systematic review^3^ showed that the most commonly used measure was the Female Sexual Function Index (FSFI).^4^ The FSFI addresses important elements of the female sexual response, but it does not address the influence of diabetes on women's sexual function.

In women with type 1 diabetes (T1D), SD is associated with diabetes-specific factors such as hypoglycaemia, diabetes technology, and the impact of diabetes on body image.^3^ These factors are not measured in the current generic FSD measures, limiting their ability to characterise SD in the lives of women with T1D. Thus, a diabetes-specific patient-reported outcome measure (PROM) of FSD is needed to provide a more valid assessment of this problem in women with T1D. Hence, we sought to develop a scale to measure FSD. However, following our exploratory work and feedback from women, we reframed the measure to sexual experience (SE), although it does cover constructs related to FSD. The word experience, which refers to the occurrence of or contact with an event, is more appropriate for describing this PROM as it has negative and positive elements within it, and it also helps avoid the medicalisation of women's sexual difficulties, allowing more focus on different factors.^5^

This paper reports the development and validation of a new measure of SE for premenopausal women with T1D. The PROM was developed to be used in research and potentially as a clinical screening measure to promote awareness of the problem in clinical consultations. We focused on premenopausal-aged women to avoid confounding factors related to the impact of menopause on women's sexual function.^6^

The study aimed to develop and test the psychometric properties (validity and reliability) of a new PROM, Female Sexual Experience in Diabetes Type 1 (FSEDiT_1), for measuring SE in premenopausal women with T1D.

## Methods

A three-phase process was used to develop the instrument and to test its validity and reliability, following the COSMIN (Consensus-based Standards for the selection of health Measurement Instrument) framework for PROM development,^7^ as follows.

• Phase 1. Identification of the constructs and items related to SE for women with T1D.
• Phase 2. Establish content validity of constructs and items.
• Phase 3. Assess the psychometric properties of FSEDiT_1 scales, measurement reliability and convergent and discriminatory validity.

This paper presents the findings from Phase 3.

### Phase 1. instrument development

In Phase 1, the conceptual framework and potential items were identified to ensure they were reflective of the SE of women with T1D. The primary data used for this phase were from a qualitative study involving premenopausal women with T1D (n = 18) who self-identified as having difficulties with sexual function.^8^ The study aimed to identify the diabetes-related factors that mediated their SE. Women with prior difficulties with sexual function were chosen so that they would be able to relay these factors. The conceptual framework was also based on the findings of a systematic review we undertook primarily to establish the prevalence of FSD in this population, but in which we were able to identify factors related to having FSD.^3^ From these data, five constructs specific to T1D were identified.^8^

• *Initiation of sexual activity*- the ability to communicate sexual interests both verbally and non-verbally.
• *Sexual confidence*- psychological and body confidence in relation to sexual activity.
• *Sexual enjoyment*- the physical and psychological pleasure of sexual activity as experienced by women with T1D.
• *Sexual engagement*- being physically and/or psychologically involved in sexual activity.
• *Sexual desire*- the ability to initiate or take part in a sexual activity.

The qualitative data from Phase 1 were also used to generate an item bank. The items were related to the constructs and were phrased with reference to the expressions and quotes from the interviews so they would be relatable to the women. Items’ content was refined and formatted with a 5-point scale, lower scores indicated diabetes-related problems in SEs (1 = always, to 5 = never).

### Phase 2. content validity

#### Focus groups

Content validity of the items compiled in Phase 1 was assessed to establish the extent to which the items reflected the construct to be measured. Items were assessed for clarity and relevance in two focus groups, one with premenopausal women with T1D and another with diabetes specialists (see Tables S1 and 2 in the Supplementary file). The participants were asked to discuss, revise, and agree on the items. They were encouraged to comment on the clarity and relevance of each item, and their comments and suggestions were reviewed by the research team, and the items were refined accordingly. The items were then compiled into a questionnaire with five subscales based on the measurement constructs.

#### Preliminary testing

Using Qualtrics, the FSEDiT_1 questionnaire was distributed electronically via social media platforms for testing. The questionnaire allowed women to comment on and provide feedback regarding the comprehensibility and acceptability of the FSEDiT_1 measure to assess its structural validity. A total of 152 responses were received. (see participants' characteristics in Tables S3 and 4 in the Supplementary file). Since the items were developed *a priori* in Phase 1, confirmatory factor analysis (CFA) was performed to assess the scaling assumptions. The items were further refined based on the women's feedback and factor loading.

#### Cognitive interviews

Cognitive interviews were conducted to assess the face and content validity of the items; and to: evaluate item comprehensibility; refine wording; eliminate ambiguities; and improve item clarity.^9^ Thirteen women were recruited via social media platforms, and three rounds of interviews were conducted (see participants’ characteristics in Table S5 in the Supplementary file). The questionnaire was sent to each participant prior to the interview. A combination of think-aloud and probing techniques was used in the interviews.^9^ The women were asked to repeat the questions in their own words and to talk about what the questions meant to them. They also commented on the relevance of items and whether the response options were applicable to them. Minor adjustments to the items were made following the interviews.

### Phase 3. psychometric testing

The psychometric properties of the FSEDiT_1 measure were then tested (see list of items used at this stage in Table S6 in the Supplementary file), considering: structural validity, internal consistency, test-retest reliability and criterion (convergent/discriminatory) validity.

#### Sample

Women with T1D were enrolled via social media platforms. Eligibility criteria included women (self-identified) aged 18–50 years, with a diagnosis of T1D for >1 year, and who were sexually active. The recommended sample size to test dimensionality and internal consistency is 10 participants for each item of the scale.^10^ As each scale was validated separately, a minimum of 340 participants was sought. However, the sample size was increased to 450 to represent different levels of education, age, and age at onset of diabetes.

#### Measures

The revised FSEDiT_1 measure, up to this phase, was the subject of the psychometric validation (see Table S6 in the Supplementary file). FSEDiT_1 is comprised of five subscales, measuring the impact of diabetes on: initiation of sexual activity (4 items), sexual confidence (7 items), sexual enjoyment (8 items), sexual engagement (8 items) and sexual desire (6 items). Each item is assessed with a 5-point scale indicating the level of SE, lower score indicating difficulties with SEs (1 = always to, 5 = never). The scores were then converted into summary scores for each subscale and a total scale score from 0 to 100, with lower scores indicating greater impact of diabetes on SE.

For the convergent and discriminatory validity testing, two additional independent measures were also incorporated into the e-survey. The *Diabetes Distress Scale* (DDS)^11^ is a 17-item scale that captures four dimensions of distress: emotional burden, regimen distress, interpersonal distress, and physician distress. It is a 6-point Likert scale from 1 (no problem) to 6 (severe problem). The original version of the DDS demonstrated reliability with Cronbach's alpha of 0·93. The DDS was used to test the convergent construct validity of the FSEDiT_1 scale.

The FSFI,^4^ is a 19-item survey designed to measure six domains of female sexual response, including desire, arousal, lubrication, orgasm, satisfaction, and pain. The summary score ranges from 2 to 36, with low scores indicating more severe SD. The FSFI showed acceptable internal consistency (Cronbach's alpha = 0·78) and acceptable test-retest reliability (Pearson correlation = 0·95). The FSFI was used for testing the discriminant validity of the FSEDiT_1 scale.

The survey also contained questions on the sociodemographic and clinical characteristics of participants, including age, age at onset of diabetes, last known glycated haemoglobin (HbA1c) level, and type of diabetes technology used.

#### Data collection

The study measures were incorporated into an e-survey using Qualtrics. A link to the questionnaire was shared via social media platforms. To prevent duplicate responses, the prevent multiple submissions function in Qualtrics was enabled, and women had to meet the eligibility criteria to access the survey. To allow the participants flexibility to review or change their responses, the function to move back to previous questions was also enabled. To enhance convenience and boost completion rates, participants could save responses and complete later.

#### Statistics

Descriptive statistics for all scales were calculated, including frequencies, percentages, means, standard deviations (SD), as well as kurtosis and skewness coefficients. Missing data were evaluated at both the variable and item levels. Sociodemographic and clinical missing data were managed using pairwise deletion, while missing responses in the scales were addressed using the Full Information Maximum Likelihood (FIML) technique, which is an appropriate method when the proportion of missing data is low.^12^

*Dimensionality* of the instruments was assessed as a preliminary step, followed by reliability testing.^13^ To examine structural validity, CFA was employed as the FSEDiT_1 scales were based on *a priori* constructs as identified in Phase 1. Factor loadings with absolute values >|0·30| were deemed satisfactory.^14^ Model fit was evaluated using several indices: Comparative Fit Index (CFI), Tucker–Lewis Index (TLI), Root Mean Square Error of Approximation (RMSEA), and Standardized Root Mean Square Residual (SRMR).^15^, ^16^, ^17^ Fit criteria were defined as follows: CFI and TLI values between 0·90 and 0·95 were considered acceptable, values > 0·95 indicated good fit^18^; RMSEA values < 0·05 and 0·05–0·08 indicated good to moderate fit respectively^19^; SRMR values < 0·08 were indicative of a good fit.^18^ Chi-square statistics were evaluated in conjunction with the fit indices. The analysis was performed separately for each of the five subscales of the FSEDiT_1.

*Reliability* was assessed using factor score determinacy^20^ and composite reliability.^21^ The global reliability indices for the multidimensional scale^22^ was computed to account for the multidimensionality of the scales.^13^ Reliability values ≥ 0·70 were deemed satisfactory.^23^

*Item discrimination* was determined by calculating the corrected item–total correlation (ITC),^24^ with values > 0·20 considered appropriate.^25^

To examine the *stability* of the FSEDiT_1 instrument, test-retest reliability was evaluated by readministering the scales after two weeks, as recommended,^26^ to 65 women selected randomly from participants who completed the initial survey using the random sample function within the Statistical Package for Social Sciences (SPSS). Intraclass Correlation Coefficients (ICCs) were computed using a two-way random effects model to analyse the scale scores. A sample of 50 participants was estimated to achieve an ICC of 0·80 (95% confidence interval [CI] ± 0·1) with two repeated measurements.^10^ Quadratic weighted kappa coefficients were calculated to assess agreement at the item level. An ICC >0·70^27^ and a weighted kappa exceeding 0·40^28^ were considered to indicate acceptable reliability.

*Convergent validity* was evaluated by assessing the hypothesis that higher diabetes distress scores would be associated with lower SE scores.^29^ The relationship between the SE (total score of each FSEDiT_1 subscale) and diabetes distress measured by DDS (total score of the distress scale) was assessed using Pearson's correlation coefficient. The cut-off for acceptable correlation was set at ≥ 0·40 (significance p < 0·05).^30^

Total FSFI and DDS scores were used in the analysis to increase data compatibility instead of applying the recommended scoring at this stage.

We verified the *construct validity* by posing several hypotheses. First, we hypothesized moderate correlations among the four scales (initiation of sexual activity, sexual enjoyment, sexual engagement and sexual desire scales) and the sexual confidence scale, as self-efficacy can be a predictor of SE. Correlation between FSEDiT_1 subscales was calculated using Pearson's product–moment correlation coefficients.

To assess the *responsiveness to change* (instrument precision), we evaluated the scale's measurement error using the standard error of measurement (SEM) and the smallest detectable change (SDC). The formula applied for SEM was the standard deviation (SD) × √(1 − reliability coefficient),^30^ where the SD represented the FSEDiT_1 scale score's SD, and the reliability coefficient was the factor score determinacy coefficient. A lower SEM value, specifically < SD/2, indicates a more precise instrument. The SDC was calculated as 1·96 × √2 × SEM.^31^ Both smaller SEM and SDC values indicate a greater instrument significance (p < 0·05). Statistical analyses were performed using SPSS Version 28, except for CFA, which was performed using Mplus Version 8·1.^20^

Each scale was assessed for dimensionality using factor analysis. The initiation of sexual activity, sexual confidence and sexual engagement scales were assessed with a single-factor solution, and the sexual enjoyment and desire scales were tested with a two-factor confirmatory model (rationale outlined below).

#### Ethics

Study Phases 1–2 were approved by the Health Research Authority/Health and Care Research/Wales (REC 22/SW/0092, dated 08/07/2022) and King's College London ethics committee (HR/DP-22/23–36186 dated 16/06/2023). Phase 3 was approved by King's College London ethics committee (HR/DP-23/24–39120 dated 22/11/2023). Informed consent was obtained from all participants.

#### Role of funding sources

The funding sources had no involvement in the conduct of this study, interpretation of findings or writing of this manuscript.

## Results

A total of 450 responses were selected randomly from 918 responses to the survey (the full dataset will be used in a subsequent analysis).

### Characteristics

A total of 429 responses were included in the analysis of this phase of the study. The mean age of the participants was 30 years (SD 8·14) and the mean age at onset of diabetes was 15·4 years (SD 8·9). The mean HbA1c was 61 mmol/L (7·7%) (SD 19·1). Almost 94% (n = 399) of participants used a continuous glucose monitoring device. Most participants identified themselves as being of White/White British ethnicity (n = 370, 86·2%). Within the UK regions, the highest percentage of participants came from Greater London (n = 67, 15·6%) and Southeast England (n = 64, 14·9%). Table 1 presents a summary of participants’ characteristics.

**Table 1:** Participants’ characteristics (Phase 3).

Number	429
Mean age (years)	30
SD	8·14
Range	18–49
Mean HbA1c	61·1 mmol (7 7%)
SD	19·1
Range	31–148
Mean age at DM onset (years)	15·4
SD	8·9
Range	1–44
Percentage using insulin pump (n)	48·9% (n = 208)
Percentage using glucose sensor (n)	93·9% (n = 399)

SD, Standard deviation; HbA1c, glycated haemoglobin; DM, diabetes mellitus.

#### Initiation of sexual activity scale

##### Dimensionality

**Fig. 1:**
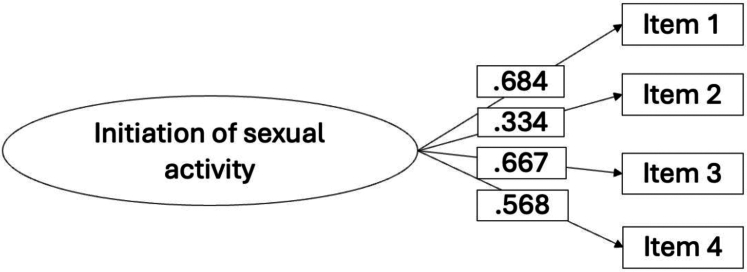
Results of the CFA for the Initiation of the Sexual Activity scale (N = 429).

The model demonstrated partially adequate fit: *χ*^2^ (2, N = 429) = 23·40, p < 0·00; CFI = 0·91, TLI = 0·75, RMSEA = 0·15 (90% CI = 0·10 0·21, p < 0 01), SRMR = 0·044. An examination of the modification indices revealed that the misfit was caused by excessive covariance between items #2 (*I avoid initiating sexual intercourse because of vaginal dryness*) and #3 (*My ability to initiate sexual activity is impacted by diabetes*). Methodological and theoretical explanations may account for this covariance. First, the proximity of these two similar items in the questionnaire may have heightened their shared meaning, thereby increasing the covariance. Secondly, vaginal dryness could make women more likely to avoid sexual activity.^32^ Specification of these residual covariances produced a final model with a good fit: *χ*^2^ (1, N = 429) = 2·25, p = 0·13; CFI = 0·99; TLI = 0·97; RMSEA = 0·05 (90% CI = 0·00 0·15, p = 0·32); SRMR = 0·01. All primary loadings were significant and presented in Table 2 and Fig. 1, the mean correlation among all items of the scale was 0·36.

**Table 2:** Factor loadings, means and standard deviations in the Initiation of Sexual Activity scale (N = 429).

Items	M (SD)	SWE (KUR)	Factor Loading
1. I avoid initiating sexual activity because I am concerned about having a hypo.	3·98 (0·90)	−0·48 (−0·29)	0·68
2. I avoid initiating sexual intercourse because of vaginal dryness.	3·87 (1·10)	−0·51 (−0·82)	0·33
3. My ability to initiate sexual activity is impacted by diabetes.	3 57 (1·09)	−0·36 (−0·59)	0·66
4. My diabetes technologies (pumps and/or sensors) make me feel anxious about initiating a sexual activity.	3·67 (1·26)	−0·53 (−0·76)	0·56

*Note*. The factor loadings estimate comes from the completely standardized solution.

SD, standard deviation; SWE, skewness; KUR, kurtosis.

##### Reliability

Factor score determinacy was 0·82 and composite reliability was 0·63. All items had adequate discrimination, presenting corrected item-correlation coefficients >0·20. The ICC coefficient for the total score of the initiation of sexual activity scale in the test-retest was 0·88 (95% CI [0·82, 0·93]). Weighted *k* varied from 0·45 to 0·59.

#### Sexual confidence scale

##### Dimensionality

**Fig. 2:**
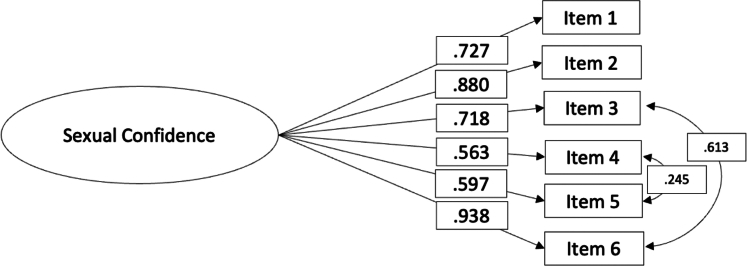
Results of the CFA for the Sexual Confidence scale (N = 429).

As two items (#6 and #3) operationalised the impact of the visibility of devices (pumps and sensors) or physical marks (bruising, scars, bleeding and lipohypertrophy) on the sexual confidence scale, we hypothesized *a priori* a correlation between their residual variances. The goodness-of-fit indices of this model was partially adequate*: χ*^*2*^ *(8, N = 429) = 38*·*69, p < 0*·*001; CFI = 0*·*96, TLI = 0*·*93, RMSEA = 0*·*09 (90% CI = 0*·*06 0*·*12, p < 0 01), SRMR = 0*·*042.* An examination of the modification indices revealed that the misfit was caused by an excessive covariance between items #4 (*I have to check my glucose levels before sexual activity in case of hypos)* and #5 (*If my glucose levels are low or going low, I lose confidence to enjoy sexual activity*). The proximity of the items in the scale increases the variance shared by these two items. In addition, these items operationalise two interrelated behaviours; checking blood glucose levels before sexual activity is related to potential hypoglycemia during intercourse. Thus, we re-ran the model and the goodness-of-fit indices of this new model were excellent: *χ*^2^ (7, N = 429) = 16·30, p = 0·02; CFI = 0·98, TLI = 0·97, RMSEA = 0·05 (90% CI = 0·02 0·09, p = 0·34), SRMR = 0·02. All primary loadings were significant (Table 3 and Fig. 2).

**Table 3:** Factor loadings, means and standard deviations of items in the Sexual Confidence scale (N = 429).

Items	M (SD)	SWE (KUR)	Factor Loading
1. I don't feel confident that I can manage changes in my glucose levels during sexual activity.	3 63 (1 15)	−0 44 (-0 58)	0 72
2. Worries about my glucose levels affect my sexual confidence.	3 66 (1 11)	−0 41 (-0 68)	0 88
3. Having visible injection/pump sites (e.g. bruising, scars, bleeding, lumps) negatively impacts on my body confidence	2 78 (1 34)	1 34 (0 22)	0 71
4. I have to check my glucose levels before sexual activity in case of hypos.	2 68 (1 34)	0 32 (-1 12)	0 56
5. If my glucose levels are low or going low, I lose confidence to enjoy sexual activity.	2 40 (1 22)	0 61 (−5 41)	0 59
6. Wearing visible diabetes technological devices (such as pumps or sensors) impacts my sexual confidence.	3 41 (1 35)	−0 34 (−1 02)	0 93

*Note*. The factor loadings estimate comes from the completely standardized solution.

SD, standard deviation; SWE, skewness; KUR, kurtosis.

##### Reliability

Factor score determinacy of the sexual confidence scale was 0·92. The Composite reliability coefficient was 0·79. The corrected item-correlation coefficients for all the items ranged from 0·46 to 0·76, showing adequate discrimination. The ICC coefficient for the total score in the test-retest was 0·87 (95% CI [0·82, 0·93]). Weighted *k* varied from 0·40 to 0·64.

#### Sexual Enjoyment scale

##### Dimensionality

**Fig. 3:**
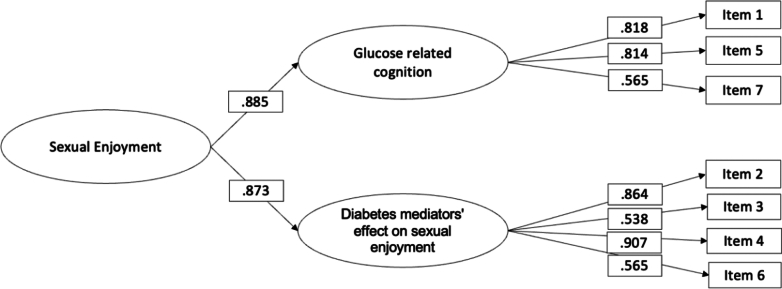
Results of the CFA for the Sexual Enjoyment scale (N = 429).

This scale was hypothesised to comprise two factors: glucose-related cognitions (F1, 3 items) and the general impact of diabetes on sexual enjoyment (F2, 4 items). The goodness-of-fit indices of this model were excellent: *χ*^2^ (13, N = 429) = 27·27, p = 0·11; CFI = 0·98, TLI = 0·98, RMSEA = 0·05 (90% CI = 0·02 0·07, p = 0·44), SRMR = 0·02. All primary loadings were significant (Table 4 and Fig. 3). Since the two factors were correlated (r = 0·77, p < 0·01), we examined a second-order hierarchical model that produced the same goodness of fit indices to the first-order model. The second-order hierarchical model shows that the sexual enjoyment scale is multidimensional at the level of primary factors, and it is unidimensional at the level of the second-order factor.

**Table 4:** Factor loadings, means and standard deviations of items in the Sexual Enjoyment scale (N = 429).

Items	M (SD)	SWE (KUR)	Factor Loading F1	Factor loading F2
1. Thinking about my glucose levels affects my enjoyment of sex.	3·38 (1·13)	−0·29 (−0·62)	0·81	
2. I experience pain/discomfort during vaginal sexual activity.	3·44 (1·10)	−0·30 (−0·49)		0·86
3. My physical sexual enjoyment (e.g. physical closeness) is negatively affected because of my diabetes.	3 78 (1·09)	−0·69 (−0·15)		0·53
4. My emotional sexual enjoyment (e.g. bonding) is negatively affected because of my diabetes.	3·83 (1·09)	−0·64 (−0·35)		0·90
5. I can't fully let go of thoughts about my diabetes to enjoy a sexual activity.	3·57 (1·23)	−0·54 (−0·70)	0·81	
6. I feel my diabetes affects my ability to orgasm.	3·72 (1·15)	−0·50 (−0·53)		0·56
7. The alarms from my diabetes devices negatively impact my sexual enjoyment.	3·14 (1·21)	−0·14 (−0·76)	0·56	

*Note*. The factor loadings estimate comes from the completely standardized solution.

SD, standard deviation; SWE, skewness; KUR, kurtosis; F1, Glucose-related cognitions; F2, Diabetes mediators' effect on sexual enjoyment.

##### Reliability

Factor scores determinacy were 0·86 and 0·92 for the glucose-related cognitions and diabetes mediators' effect on sexual enjoyment factors. The factor score determinacy was 0·86 for the sexual enjoyment dimension. The composite reliability scores were 0·78, 0·82 for the glucose-related cognitions and diabetes mediators' effect on sexual enjoyment, respectively. The global reliability indices for the multidimensional scale was 0·83. All items presented adequate discrimination, with ITCs higher than 0·20. The ICC coefficient for the total score in the test-retest was 0·94 (95% CI [0·92, 0·97]). Weighted *k* varied from 0·47 to 0·60.

#### Sexual engagement scale

##### Dimensionality

**Fig. 4:**
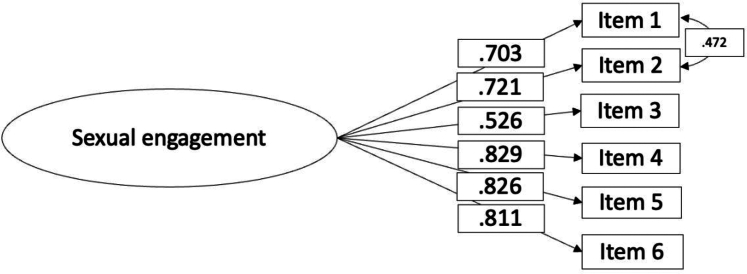
Results of the CFA for the Sexual Engagement scale (N = 429).

As two items were based on complementary propositions, we anticipated *a priori*, excess shared variance between these two items. Therefore, residual covariance between items #1 (*My glucose levels affect how I physically engage in sexual activity)* and #2 *(My glucose levels affect how I emotionally engage in sexual activity)* was specified. This model had good fit: *χ*^2^ (8, N = 429) = 29·85, p < 0 01; CFI = 0·98, TLI = 0·97, RMSEA = 0·08 (90% CI = 0·05 0·11, p = 0·04), SRMR = 0·02. All factor loadings are significant and presented in Table 5 and Fig. 4.

**Table 5:** Factor loadings, means and standard deviations of items in the Sexual Engagement scale (N = 429).

Items	M (SD)	SWE (KUR)	Factor Loading
1. My glucose levels affect how I physically engage (e.g. physical closeness) in sexual activity.	3·37 (1·12)	−0·26 (−0·55)	0·70
2. My glucose levels affect how I emotionally engage (e.g. bond) in sexual activity.	3·57 (1·13)	−0·40 (−0·59)	0·72
3. I stop engaging in sexual activity if I am fearful of having a hypo.	3·02 (1·24)	0·01 (1·00)	0·52
4. I find engaging in sexual activity difficult because of my diabetes.	3·76 (1·05)	−0·56 (−0·37)	0·82
5. I have to make extra efforts to engage in sexual activity because of my diabetes.	3·62 (1·19)	−0·47 (−0·76)	0·82
6. There is a lack of spontaneity in sexual activity because of my diabetes.	3·45 (1·28)	−0·41 (0·85)	0·81

*Note*. The factor loadings estimate comes from the completely standardized solution.

SD, standard deviation; SWE, skewness; KUR, kurtosis.

##### Reliability

The factor score determinacy was 0·94, and the composite reliability coefficient was 0·88. All items presented adequate discrimination, with ITCs >0·20. The ICC coefficient for the total score in the test-retest was 0·94 (95% CI [0·92, 0·97]). Weighted *k* varied from 0·40 to 0·64.

#### Sexual desire scale

##### Dimensionality

**Fig. 5:**
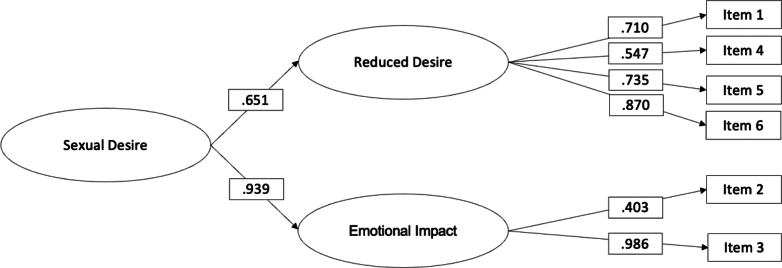
Results of the CFA for the Sexual Desire scale (N = 429).

This scale was hypothesised to be comprised of two factors: Reduced Desire (F1, 4 items) and Emotional Impact (F2, 2 items). The goodness-of-fit indices of this model were excellent: *χ*^2^ (8, N = 429) = 15·89, p = 0·04; CFI = 0·99, TLI = 0·98, RMSEA = 0·04 (90% CI = 0·00 0·08, p = 0·48), SRMR = 0·02. All primary loadings were significant (Table 6 and Fig. 5). Since the two factors were correlated (r = 0·61, p < 0·00), we examined a second-order hierarchical model that produced the same goodness-of-fit indices to the first-order model. The second-order hierarchical model shows that the sexual desire scale is multidimensional at the level of primary factors, and it is unidimensional at the level of the second-order factor.

**Table 6:** Factor loadings, means and standard deviations of items in the Sexual Desire scale (N = 429).

Items	M (SD)	SWE (KUR)	Factor Loading F1	Factor Loading F2
1. I lack sexual desire because of my diabetes.	3·54 (1·17)	−0·27 (−0 87)	0·71	
2. My mood is affected by having diabetes, and this reduces my sexual desire.	2·92 (1·13)	0·08 (−0·54)		0·40
3. I worry about getting pregnant with diabetes and this impacts my sexual desire.	3·61 (1·35)	−0·59 (−0·84)		0·98
4. I lack energy because of diabetes, and this impacts my sexual desire.	2·82 (1·09)	0 25 (−0·51)	0·54	
5. I feel I let my intimate partner(s) down because diabetes affects my sexual desire.	3·21 (1·32)	−0·10 (−1·12)	0·73	
6. In moments of intimacy with my partner(s), I don't feel sexually aroused because of my diabetes.	3·70 (1·10)	−0·57 (−0·27)	0·87	

*Note*. The factor loadings estimate comes from the completely standardized solution.

SD, standard deviation; SWE, skewness; KUR, kurtosis; F1, Reduced desire; F2, Emotional impact.

##### Reliability

Factor scores determinacy for the Reduced Desire and Emotional Impact factors were 0·92 and 0·98, respectively. Factor scores determinacy for the sexual desire dimension was 0·93. Composite reliability for the Reduced Desire and Emotional Impact were 0·82 and 0·70, respectively. The global reliability indices for the multidimensional scale was 0·83. The corrected item-correlation coefficients for all the items were >0·20, demonstrating adequate discrimination. The ICC coefficient for the total score in the test-retest was 0·93 (95% CI [0·89, 0·96]). Weighted *k* varied from 0·50 to 0·62.

### Convergent validity

For convergent validity, there was, as expected, a significant (p < 0·01) negative correlation between the total score of the subscales and the total score of the DDS (−0·45), supporting the construct validity of FSEDiT_1.

### Discriminant validity

There was a small but significant negative correlation between the total score of the subscales and the total score of the FSFI (−0·14, p < 0·01), suggesting that the two scales are distinct in what they measure, with a small measurement overlap.

### Construct validity

The correlation between the sexual confidence score and the initiation of sexual activity, sexual enjoyment, sexual engagement and sexual desire scores ranged from 0·50 to 0·69.

### Measurement error

The SEM of the SD on the scales was adequate, as follows: 11.60 for initiation of sexual activity; 9·90 for Sexual confidence; 8 95 for sexual enjoyment; 8·11 for sexual engagement; and 9·45 for sexual desire. The SDC was: 9·44 for initiation of sexual activity; 8·72 for sexual confidence; 8·15 for sexual enjoyment; 7·89 for sexual engagement; and 8·52 for sexual desire.

Figures S1-6 in the Supplementary file show the distribution of the scales, and Table S7 shows the Mean (SD) of the scores.

## Discussion

This study, following a rigorous approach based on current guidelines for PROM development^7^ has demonstrated that FSEDiT_1 is a theoretically grounded, valid and reliable tool for measuring SEs in premenopausal women with T1D. The FSEDiT_1 measure is the first diabetes-specific measure for assessing female SE. A key strength of this measure is that the scale items are based on the experiences of women with T1D.

The final list of items can be found in Table S8 in the Supplementary file. During the analysis, one item (*Diabetes has an impact on how confident I feel in expressing sexual interes*t) was deleted from the sexual confidence scale due to non-significant factor loading. It could be that this item was too broad, not specifying different aspects of diabetes. However, during the cognitive interviews, most women found this item to be clear. It is possible that its phrasing, which combines two elements, one addressing sexual confidence and the other sexual interest, may have influenced how the item performed psychometrically. Another item (*I develop genital thrush, which affects my enjoyment of sex*) was deleted from the sexual enjoyment scale as it presented a low factor loading. This item might be different as it expresses an intermittent problem, whereas other items in this scale describe continuous emotions and feelings. During the qualitative interviews in Phase 1, one participant said, *“My blood sugars aren't that high continuously, so occasionally I might get that* (referring to genital thrush)” (Participant 1, aged 44). Another participant said, “*I used to get it all the time, but then as soon as I got diagnosed 25 years ago, I never had thrush*” (participant 4, aged 47). Although the prevalence of vulvovaginal conditions is higher in women with T1D,^33^ it could be that the use of diabetes technology and the resulting improvement in glycaemic outcomes may have helped reduce the impact of these conditions.

Two items were deleted from the sexual engagement scale (*I check my glucose levels during sexual activities; I stop engaging in sexual activity if I become aware that I am experiencing a hypo)* due to low factor loading. Women may check their glucose levels before or after, not during, sexual activity. The second item may need to be more specified in terms of the type of sexual activity. This question was the last in the engagement scale. It is possible that this question may have performed differently if it had been repositioned in the scale. No issues were identified with these items during the cognitive interviews.

The subscales of the measure provide independent perspectives on diabetes-specific impacts on SEs, these include: wearable diabetes technologies, body image issues, anxiety about hypoglycaemia, and the physical impact of diabetes on SEs. Hence, the FSEDiT_1 measure could be used to inform clinical care and the development and evaluation of supportive interventions to help women address and minimise these impacts. FSD is a common problem (odds ratio = 3·8 95% CI 1·8–8·0, p < 0·00^3^), that is often an overlooked topic in clinical care and is under-researched.^34^ FSEDiT_1 could provide a stimulus for more research into this neglected complication of diabetes. It could be used clinically to highlight women's concerns relating to sexual intimacy. The high rate of completion and the small number of missing data suggest that the measure is acceptable to women with T1D. The proportion of missing data was relatively low in this study, with close to 90% of the responses having no missing data. This is true for the three measures used in this phase of the study. Within the 10% margin that includes a large part of missing data, there was a question for which participants were given the option not to complete the question because they may or may not be using particular technologies. Data from the preliminary testing suggests that the average time to completion was 14 min.

To enhance the utility of the measure, we plan a subsequent analysis of the full dataset (n = 918) to assess variables associated with FSEDiT_1 to establish cut-off scores.

There may be an ethnic bias in the study in that the majority of participants were of White British ethnicity. While this could have been related to the general low participation of people from ethnic minorities in research,^35^ socioeconomic factors,^36^ and problems with internet access,^37^ it is more likely related to the higher prevalence of T1D in people of White ethnicity in the UK. The most recent national diabetes audit data for the UK show that 85% of the T1D population is of White ethnicity.^38^ Hence, validating the measure in country contexts or languages would help enhance the generalisability of the FSEDiT_1 measure.

There was also a potential age bias in that only 15% (n = 66) of participants were aged >40 years. The most likely explanation for this bias is that the younger women were more motivated, as they are likely to be more sexually active and may still be in the formative stage of developing relationships. A recent large UK survey reported that sexual activity is higher in younger people and declines significantly after the age of 35 years.^39^

Finally, it is important to acknowledge the challenges related to data reliability in online surveys, particularly the risk of fraudulent responses and survey bots.^37^ To mitigate these risks, this study implemented several quality control measures. These include adding a CAPTCHA (Completely Automated Public Turing test to tell Computers and Humans Apart) to distinguish human participants from automated bots, as well as routine data checks to ensure the quality of the responses.

Future studies are needed to establish the clinical utility of this PROM. There are several levels at which this can be considered, such as addressing questions related to the variation in the SE of women in relation to the duration of diabetes. Another question is related to the impact of diabetes technology on women's SE. This RPOM could also be used in an exploratory study to trigger a conversation about the SE in current clinical practice. Importantly, future studies are needed to evaluate this PROM in more diverse populations, in countries outside the UK. Ultimately, we intend to develop clinical cut-off values to estimate the magnitude of impact expressed in the different scales, both conceptually and empirically.

FSEDiT_1 presents a valid and reliable measure that could be used in research to improve our understanding of the sexual issues experienced by women with T1D. While there was rigorous work completed to develop this tool, additional psychometric testing is necessary before it can be used in a clinical setting to inform decision-making. Potentially, the measure could be used clinically to: raise the profile of women's SEs and challenges, improve the support they receive, and inform the development of care pathways and management strategies related to SEs (sexual: initiation, confidence, enjoyment, engagement and desire).

## Contributors

All authors contributed to the study design and provided critical input. RH collected and managed the data. RH, MDM and AF had direct access to the data. RH, MDM and AF have verified the underlying data. Data analysis conducted by RH, MDM and AF. RH wrote the first draft of the manuscript, which was critically reviewed and revised by all authors. All authors read and approved the final version.

## Data sharing statement

The dataset used in this study is available from the corresponding author upon reasonable request.

## Declaration of interests

Novo Nordisk supported RH to attend a professional conference. Other authors have no declaration of interest to declare.
