# Supplementary material for: Sexual experience in premenopausal women with type 1 diabetes: development and validation of a patient-reported outcome measure (PROM)
Source: eClinicalMedicine. 2025 Oct 30;90:103608. doi: 10.1016/j.eclinm.2025.103608 (PMC12615296; doi:10.1016/j.eclinm.2025.103608)
Supplement: Supplementary Material [file mmc1.docx]

**Supplementary materials**

**Table of contents**

| **Content** | **Page** |
| --- | --- |
| Table 1: Focus Group 1 participants' characteristics | 2 |
| Table 2: Focus Group 2 participants' characteristics | 2 |
| Table 3: Demographic data/ Preliminary testing | 3 |
| Table 4: Ethnicity/ preliminary testing | 3 |
| Table 5: Cognitive interviews participants' characteristics | 4 |
| Table 6: Questionnaire used in the psychometric study | 5 |
| Figure 1: Initiation of sexual activity scale scores distribution | 7 |
| Figure 2: Sexual confidence scale scores distribution | 7 |
| Figure 3: Sexual enjoyment scale score distribution | 8 |
| Figure 4: Sexual engagement scale score distribution | 8 |
| Figure 5: Sexual desire scale score distribution | 9 |
| Figure 6: Total FSEDiT_1 scores distribution | 9 |
| Table 7: Mean and Std. Deviation results of all scales | 10 |
| Table 8: FSEDiT_1 final item list | 10 |

**Table 1: Focus Group 1- women with type 1 characteristics**

| **Participant number** | **Age** | **Age of onset of diabetes** | **Length of current relationship/ years** | **Number of children** | **Number of pregnancies** | **Diabetes technology/ insulin delivery** | **Latest HbA1c mmol/mol (%)** | **Occupation** |
| --- | --- | --- | --- | --- | --- | --- | --- | --- |
| **1** | 34 | 31 | 2.5 | 0 | 12 | CGM/ MDI | 60 (7.6) | Receptionist |
| **2** | 38 | 21 | 9 | 0 | 1 | CGM/ MDI | 51 (6.8) | Production team leader |
| **3** | 28 | 7 | NA | 0 | 0 | CGM  Insulin pump | 53 (7) | Project manager |
| **4** | 42 | 5 | 15 | 2 | 2 | CGM/ MDI | 85 (9.9) | Housekeeper |
| CGM= continuous glucose monitor  MDI= multiple daily injections  HbA1c= Glycated haemoglobin | | | | | | | | |

**Table 2: Focus Group 2- Diabetes health professionals characteristics**

| **Member** | **Age** | **Occupation** | **Years of experience** | **Diabetes qualification** | **Knowledge about FSD from previous education Y/N** | **Pathway for treatment of FSD in service provision Y/N** |
| --- | --- | --- | --- | --- | --- | --- |
| 1 | 42 | Lead DSN | 10 | MSc | N | N |
| 2 | 36 | Diabetes clinical psychologist | 5.5 | NA | N | N |
| 3 | 42 | Diabetes practice nurse | 11 | Diabetes course | N | N |
| 4 | 51 | DSN | 3 | Diabetes certificate | N | N |
| 5 | 36 | Lead DSN | 10 | MSc | Y | N |

**Table 3: Demographic data/ Preliminary testing**

| Characteristic | N | Minimum | Maximum | Mean | Std. Deviation |
| --- | --- | --- | --- | --- | --- |
| Age | 152 | 21 | 48 | 34.77 | 7.072 |
| Age of onset of diabetes | 147 | 1 | 45 | 17.92 | 10.864 |
| Last known HbA1c | 121 | 31 | 140 | 56.32 | 17.914 |

**Table 4: Ethnicity/ preliminary testing**

| Valid | Frequency | Percent |
| --- | --- | --- |
| Other | 2 | 1.4 |
| Asian | 5 | 3.3 |
| Black | 7 | 4.6 |
| Hispanic | 1 | .7 |
| Latin | 1 | .7 |
| Mixed | 6 | 3.9 |
| White | 129 | 84.9 |
| Chinese | 1 | .7 |
| Total | 152 | 100.0 |

**Table 5: Cognitive interview characteristics**

| **ID** | **Age** | **Age of onset of diabetes** | **Duration of diabetes** | **Education level** | **Married or in a relationship**  **Y/N** | **Number of pregnancies** | **Number of children** | **Diabetes technology** |
| --- | --- | --- | --- | --- | --- | --- | --- | --- |
| 01 | 44 | 17 | 27 | Batchelor’s degree | Y | 0 | 0 | CGM |
| 02 | 45 | 4 | 41 | Bachelor’s degree | Y | 4 | 1 | HCL |
| 03 | 31 | 11 | 20 | Graduate | Y | 3 | 1 | HCL |
| 04 | 47 | 22 | 25 | Bachelor’s degree | N | 1 | 1 | NA |
| 05 | 43 | 32 | 11 | NR | Y | 2 | 2 | HCL |
| 06 | 42 | 7 | 35 | Master’s degree | Y | 0 | 0 | HCL |
| 07 | 48 | 4 | 44 | Diploma | Y | 2 | 2 | HCL |
| 08 | 42 | 10 | 32 | Bachelor’s degree | Y | 3 | 3 | HCL |
| 09 | 44 | 12 | 32 | University student | Y | 3 | 2 | HCL |
| 10 | 26 | 11 | 15 | Master’s degree | N | 0 | 0 | CGM |
| 11 | 36 | 9 | 27 | Master’s degree | Y | 1 | 1 | HCL |
| 12 | 44 | 7 | 37 | Master’s degree | Y | 0 | 0 | HCL |
| 13 | 20 | 7 months | 20 | University student | Y | 0 | 0 | HCL |
| CGM= Continuous glucose monitoring; HCL= Hybrid closed loop; NA= non-applicable; NR= Not reported | | | | | | | | |

**Table 6: Questionnaire used in the psychometric testing**

| **Item** | **Always**  **1** | **Most of the time**  **2** | **Occasionally**  **3** | **Rarely**  **4** | **Never**  **5** |
| --- | --- | --- | --- | --- | --- |
| Subscale 1: Initiation of sexual activity  Initiation of sexual activity refers to whether living with type 1 diabetes impacts your confidence or interest in wanting to initiate sexual activity. There are 4 questions in this part. Please select your answer from the dropdown list. | | | | | |
| 1- I avoid initiating sexual activity because I am concerned about having a hypo. |  |  |  |  |  |
| 2- I avoid initiating sexual intercourse because of vaginal dryness. |  |  |  |  |  |
| 3- My ability to initiate sexual activity is impacted by diabetes. |  |  |  |  |  |
| 4- Only answer if you are using diabetes technology.  My diabetes technologies (pumps and/or sensors) make me feel anxious about initiating a sexual activity. |  |  |  |  |  |
| **Subscale 2: Sexual confidence**  In the context of this questionnaire, female sexual confidence is defined as having the confidence and ability to express sexual interest and being comfortable within one’s own body in the presence of challenges related to living with type 1 diabetes. There are 7 questions in this section. Please select your answer from the dropdown list. | | | | | |
| 1- I don't feel confident that I can manage changes in my glucose levels during sexual activity. |  |  |  |  |  |
| 2- Worries about my glucose levels affect my sexual confidence. |  |  |  |  |  |
| 3- Diabetes has an impact on how confident I feel in expressing sexual interest. |  |  |  |  |  |
| 4- Having visible injection/pump sites (e.g. bruising, scars, bleeding, lumps) negatively impacts on my body confidence |  |  |  |  |  |
| 5- I have to check my glucose levels before sexual activity in case of hypos. |  |  |  |  |  |
| 6- If my glucose levels are low or going low, I lose confidence to enjoy sexual activity |  |  |  |  |  |
| 7-Only answer if you are using diabetes technology.  Wearing visible diabetes technological devices (such as pumps or sensors) impacts my sexual confidence. |  |  |  |  |  |
| **Subscale 3: Sexual enjoyment**  Sexual enjoyment refers to the physical and/or psychological pleasure experienced by women with type 1 diabetes during sexual activity. There are 8 questions in this part. | | | | | |
| 1- Thinking about my glucose levels affects my enjoyment of sex |  |  |  |  |  |
| 2- I develop genital thrush which affects my enjoyment of sex |  |  |  |  |  |
| 3- I experience pain/discomfort during vaginal sexual activity. |  |  |  |  |  |
| 4- My physical sexual enjoyment (e.g. physical closeness) is negatively affected because of my diabetes. |  |  |  |  |  |
| 5- My emotional sexual enjoyment (e.g. bonding) is negatively affected because of my diabetes. |  |  |  |  |  |
| 6- I can’t fully let go of thoughts about my diabetes to enjoy a sexual activity. |  |  |  |  |  |
| 7- I feel my diabetes affects my ability to orgasm. |  |  |  |  |  |
| 8- Only answer if you are using diabetes technology with alarms.  The alarms from my diabetes devices negatively impact my sexual enjoyment |  |  |  |  |  |
| **Subscale 4: Sexual engagement**  Sexual engagement refers to the extent to which having type 1 diabetes can impact how you engage in or experience (physically and/or psychologically) sexual activity. There are 8 questions in this section. | | | | | |
| 1- My glucose levels affect how I physically engage (e.g. physical closeness) in sexual activity. |  |  |  |  |  |
| 2- My glucose levels affect how I emotionally engage (e.g. bond) in sexual activity |  |  |  |  |  |
| 3- I stop engaging in sexual activity if I am fearful of having a hypo |  |  |  |  |  |
| 4- I find engaging in sexual activity difficult because of my diabetes. |  |  |  |  |  |
| 5- I have to make extra efforts to engage in sexual activity because of my diabetes |  |  |  |  |  |
| 6- There is a lack of spontaneity in sexual activity because of my diabetes |  |  |  |  |  |
| 7- I check my glucose levels during sexual activities |  |  |  |  |  |
| 8-I stop engaging in sexual activity if I become aware that I am experiencing a hypo |  |  |  |  |  |
| **Subscale 5: Sexual desire**  Sexual desire refers to the willingness or interest of women with type 1 diabetes to initiate and/or take part in a sexual activity. There are 6 questions in this section. | | | | | |
| 1-I lack sexual desire because of my diabetes. |  |  |  |  |  |
| 2- My mood is affected by having diabetes, and this reduces my sexual desire |  |  |  |  |  |
| 3- I worry about getting pregnant with diabetes, and this impacts my sexual desire. |  |  |  |  |  |
| 4- I lack energy because of diabetes, and this impacts on sexual desire. |  |  |  |  |  |
| 5- I feel I let my intimate partner(s) down because diabetes affects my sexual desire |  |  |  |  |  |
| 6- In moments of intimacy with my partner(s), I don't feel sexually aroused because of my diabetes. |  |  |  |  |  |

**Figure 1: Distribution of scores- Initiation of sexual activity scale**


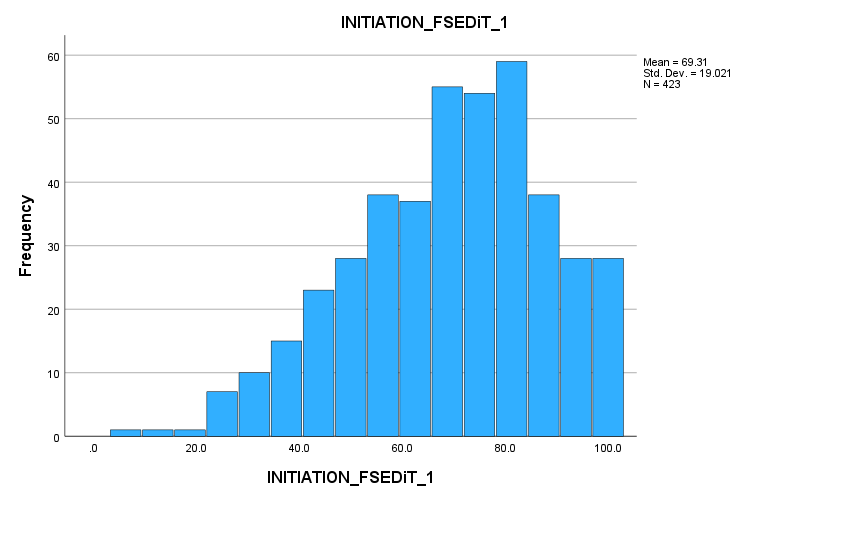


**Figure 2: Distribution of scores: Sexual confidence scale**


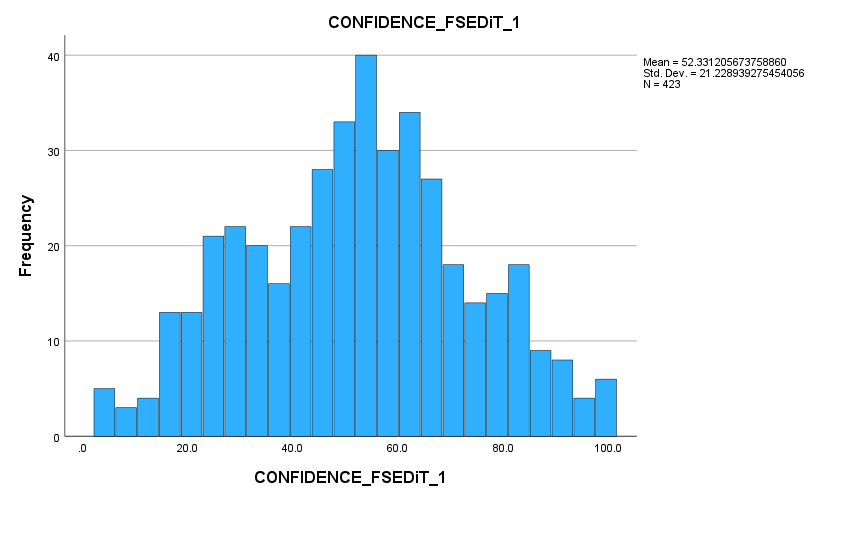


**Figure 3: Distribution of scores: Sexual enjoyment scale**


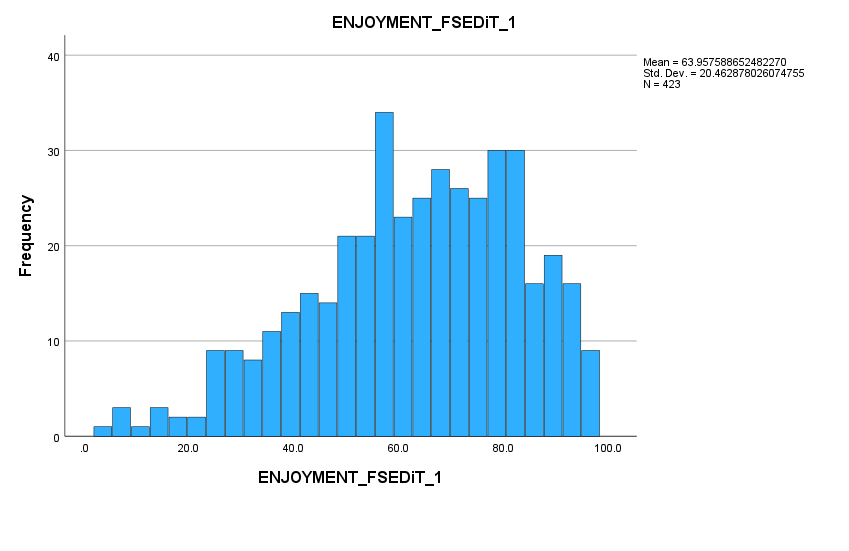


**Figure 4: Distribution of scores: Sexual engagement scale**


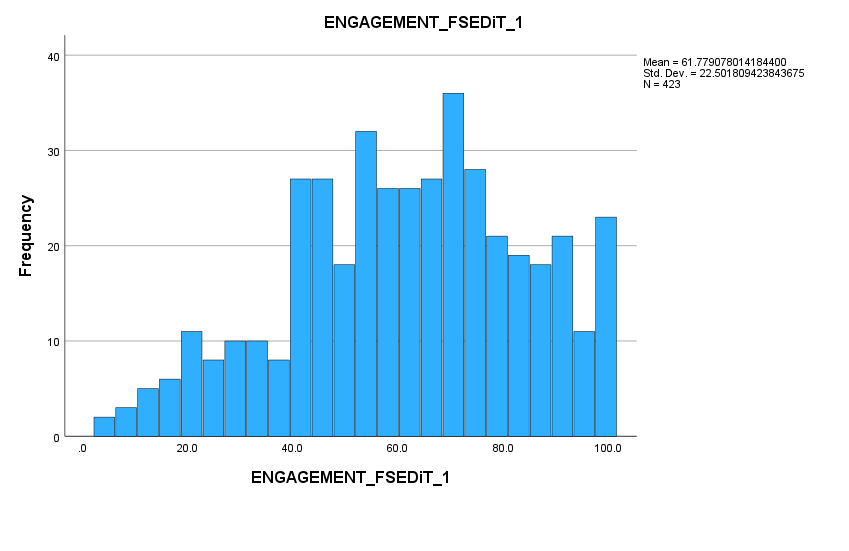


**Figure 5: Distribution of scores: Sexual desire scale**

**
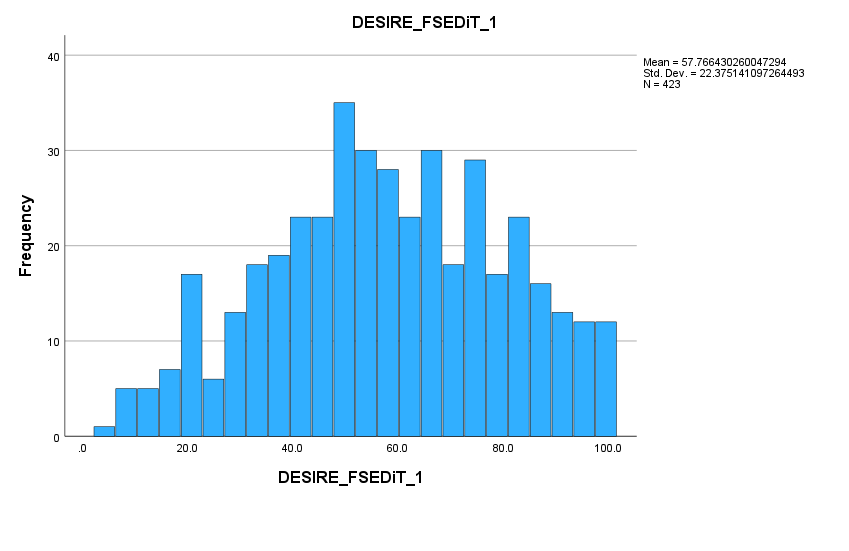
**

**Figure 6: FSEDiT_1 scores distribution**


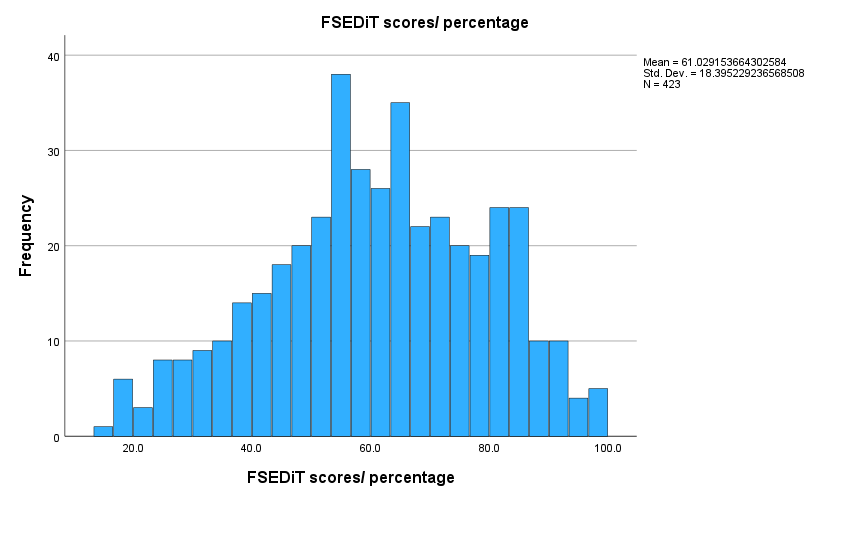


**Table 7: Mean and Std. Deviation results of all scales**

|  | Initiation FSEDiT | Confidence  FSEDiT | Enjoyment  FSEDiT | Engagement  FSEDiT | Desire  FSEDiT | FSEDiT scores/percentages | FSFI scores | DDS scores |
| --- | --- | --- | --- | --- | --- | --- | --- | --- |
| N Valid | 423 | 423 | 423 | 423 | 423 | 423 | 423 | 423 |
| Mean | 69.31 | 52.33 | 63.95 | 61.77 | 57.76 | 61.02 | 25.61 | 110.51 |
| Std. Deviation | 19.02 | 21.22 | 20.46 | 22.50 | 22.37 | 18.39 | 4.78 | 37.822 |

**Table 8: FSEDiT_1 scale, final item list following assessment of the psychometric properties**

| **Item** | **Always**  **1** | **Most of the time**  **2** | **Occasionally**  **3** | **Rarely**  **4** | **Never**  **5** |
| --- | --- | --- | --- | --- | --- |
| **Scale 1: Initiation of sexual activity**  Initiation of sexual activity refers to whether living with type 1 diabetes impacts your confidence or interest in wanting to initiate sexual activity. There are 4 questions in this scale. | | | | | |
| 1- I avoid initiating sexual activity because I am concerned about having a hypo. |  |  |  |  |  |
| 2- I avoid initiating sexual intercourse because of vaginal dryness. |  |  |  |  |  |
| 3- My ability to initiate sexual activity is impacted by diabetes. |  |  |  |  |  |
| 4- Only answer if you are using diabetes technology.  My diabetes technologies (pumps and/or sensors) make me feel anxious about initiating a sexual activity. |  |  |  |  |  |
| **Scale 2: Sexual confidence**  In the context of this questionnaire, female sexual confidence is defined as having the confidence and ability to express sexual interest and being comfortable within one’s own body in the presence of challenges related to living with type 1 diabetes. There are 6 questions in this scale. | | | | | |
| 1- I don't feel confident that I can manage changes in my glucose levels during sexual activity. |  |  |  |  |  |
| 2- Worries about my glucose levels affect my sexual confidence. |  |  |  |  |  |
| 3- Having visible injection/pump sites (e.g. bruising, scars, bleeding, lumps) negatively impacts on my body confidence. |  |  |  |  |  |
| 4- I have to check my glucose levels before sexual activity in case of hypos. |  |  |  |  |  |
| 5- If my glucose levels are low or going low, I lose confidence to enjoy sexual activity. |  |  |  |  |  |
| 6- Only answer if you are using diabetes technology.  Wearing visible diabetes technological devices (such as pumps or sensors) impacts my sexual confidence. |  |  |  |  |  |
| **Scale 3: Sexual enjoyment**  Sexual enjoyment refers to the physical and/or psychological pleasure experienced by women with type 1 diabetes during sexual activity. There are 7 questions in this scale. | | | | | |
| 1- Thinking about my glucose levels affects my enjoyment of sex. |  |  |  |  |  |
| 2- I experience pain/discomfort during vaginal sexual activity. |  |  |  |  |  |
| 3- My physical sexual enjoyment (e.g. physical closeness) is negatively affected because of my diabetes. |  |  |  |  |  |
| 4- My emotional sexual enjoyment (e.g. bonding) is negatively affected because of my diabetes. |  |  |  |  |  |
| 5- I can’t fully let go of thoughts about my diabetes to enjoy sexual activity. |  |  |  |  |  |
| 6- I feel my diabetes affects my ability to orgasm. |  |  |  |  |  |
| 7- Only answer if you are using diabetes technology with alarms.  The alarms from my diabetes devices negatively impact my sexual enjoyment. |  |  |  |  |  |
| **Scale 4: Sexual engagement**  Sexual engagement refers to the extent to which having type 1 diabetes can impact how you engage in or experience (physically and/or psychologically) sexual activities. There are 6 questions in this scale. | | | | | |
| 1-My glucose levels affect how I physically engage (e.g. physical closeness) in sexual activity. |  |  |  |  |  |
| 2-My glucose levels affect how I emotionally engage (e.g. bond) in sexual activity. |  |  |  |  |  |
| 3-I stop engaging in sexual activity if I am fearful of having a hypo |  |  |  |  |  |
| 4-I find engaging in sexual activity difficult because of my diabetes. |  |  |  |  |  |
| 5-I have to make extra efforts to engage in sexual activity because of my diabetes. |  |  |  |  |  |
| 6-There is a lack of spontaneity in sexual activity because of my diabetes. |  |  |  |  |  |
| **Scale 5: Sexual desire**  Sexual desire refers to the willingness or interest of women with type 1 diabetes to initiate and/or take part in a sexual activity. There are 6 questions in this scale. | | | | | |
| 1-I lack sexual desire because of my diabetes. |  |  |  |  |  |
| 2-My mood is affected by having diabetes, and this reduces my sexual desire. |  |  |  |  |  |
| 3-I worry about getting pregnant with diabetes and this impacts my sexual desire. |  |  |  |  |  |
| 4-I lack energy because of diabetes, and this impacts my sexual desire. |  |  |  |  |  |
| 5-I feel I let my intimate partner(s) down because diabetes affects my sexual desire. |  |  |  |  |  |
| 6-In moments of intimacy with my partner(s), I don't feel sexually aroused because of my diabetes. |  |  |  |  |  |

**©Copyright 2025 King’s College London**

**Kindly inform one of the authors if you wish to use this questionnaire.**
